# The nuclear transportation routes of membrane-bound transcription factors

**DOI:** 10.1186/s12964-018-0224-3

**Published:** 2018-04-03

**Authors:** Yang Liu, Peiyao Li, Li Fan, Minghua Wu

**Affiliations:** 1 Hunan Provincial Tumor Hospital and the Affiliated Tumor Hospital of Xiangya Medical School, Central South University, Changsha, 410013 Hunan China; 2 The Key Laboratory of Carcinogenesis of the Chinese Ministry of Health, The Key Laboratory of Carcinogenesis and Cancer Invasion of the Chinese Ministry of Education, Cancer Research Institute, Central South University, Changsha, 410008 Hunan China; 3 Department of Biochemistry, University of California, Riverside, CA 92521 USA

## Abstract

Membrane-bound transcription factors (MTFs) are transcription factors (TFs) that are anchored in membranes in a dormant state. Activated by external or internal stimuli, MTFs are released from parent membranes and are transported to the nucleus. Existing research indicates that some plasma membrane (PM)-bound proteins and some endoplasmic reticulum (ER) membrane-bound proteins have the ability to enter the nucleus. Upon specific signal recognition cues, some PM-bound TFs undergo proteolytic cleavage to liberate the intracellular fragments that enter the nucleus to control gene transcription. However, lipid-anchored PM-bound proteins enter the nucleus in their full length for depalmitoylation. In addition, some PM-bound TFs exist as full-length proteins in cell nucleus via trafficking to the Golgi and the ER, where membrane-releasing mechanisms rely on endocytosis. In contrast, the ER membrane-bound TFs relocate to the nucleus directly or by trafficking to the Golgi. In both of these pathways, only the fragments of the ER membrane-bound TFs transit to the nucleus. Several different nuclear trafficking modes of MTFs are summarized in this review, providing an effective supplement to the mechanisms of signal transduction and gene regulation. Moreover, targeting intracellular movement pathways of disease-associated MTFs may significantly improve the survival of patients.

## Background

Gene expression is controlled by specific interactions between transcription factors, regulatory proteins, and cis-elements in the gene regulatory regions [1]. Existing research shows that transcription factors are not only proteins but numerous non-coding RNAs act as regulators of transcription [2]. Many long non-coding RNAs have been found to play important roles in the regulation of gene expression [3]. Most transcription factors are located in the cytoplasm. After receiving a signal from the cell membrane signal transduction, transcription factors are activated and then translocated from the cytoplasm into the nucleus where they interact with the corresponding DNA frame (cis-acting elements). A transcription factor usually has one or more DNA-binding domains, and therefore can regulate the expression of multiple genes. Conversely, one gene can be regulated by many transcription factors.

Some membrane proteins play a transcription regulatory role after being translocated into the nucleus. Membrane-bound transcription factors (MTFs) have been observed in many types of organisms, such as plants, animals and microorganisms [4–6]. According to the activation routes of general transcription factors, these molecules are regulated at many points throughout signal transduction in an exquisite process. As a result, membrane-bound transcription factors can respond rapidly to stresses from either extracellular or intracellular stimuli [7]. Full-length MTFs are synthesized in the cytoplasm and are rapidly transported to the cellular membrane [8]. Once they are anchored in the cell membrane, MTFs remain in a dormant state [7, 9]. Cellular stimuli can activate transcription factor precursors and induce their nuclear translocation. Nuclear translocation signals that have been established include ligand-receptor binding response signals as well as many types of stress, such as nerve injury stress, temperature stress, endoplasmic reticulum (ER) stress, and oxidative stress, among others [10–14]. In this review, we mainly focus on how MTFs move within the cell and whether they turn to other organelle membranes during their nuclear translocation. We separate MTFs into two groups: plasma membrane-bound (PM) transcription factors and ER membrane-bound transcription factors. PM proteins are translocated into the nucleus directly or by Golgi to ER retrograde trafficking, either in their full-length or in their cytosolic fragmented form. ER membrane-bound proteins are translocated into the nucleus directly or by trafficking first to the Golgi from the ER, but both routes give rise to the fragments of the proteins finally entering the nucleus.

## The nuclear transportation routes of plasma membrane-bound proteins

### Plasma membrane-bound proteins are translocated into the nucleus directly

#### RIP-dependent release from the plasma membrane

**Fig. 1:**
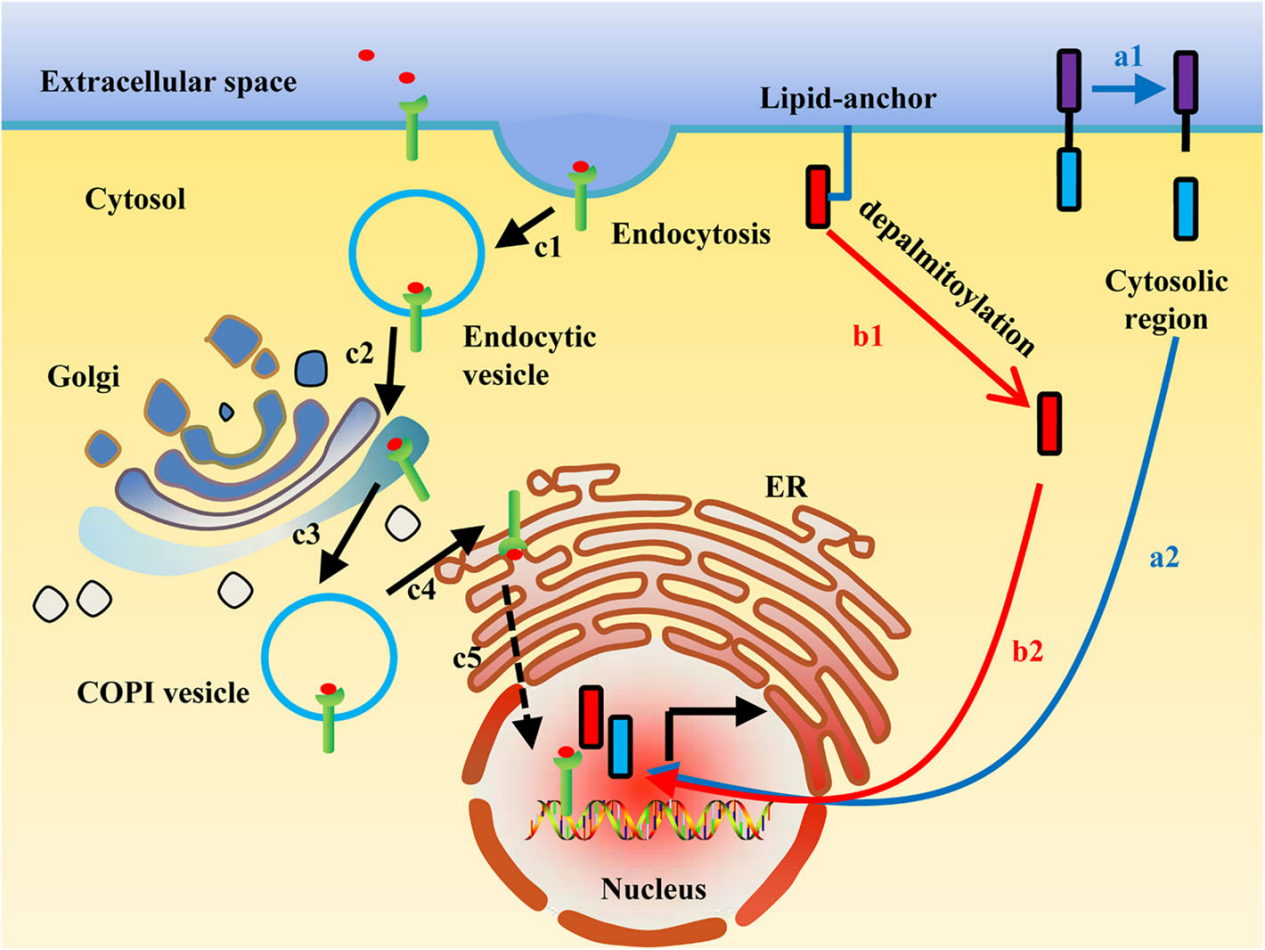
The nuclear transportation routes of plasma membrane-bound proteins. (a1-a2) Some PM proteins are cleaved by regulated intramembrane proteolysis. The fragments facing to cytosol are released and relocated to the nucleus. (b1-b2) Some of them attach to the plasma membrane by palmitoylation. They release from the plasma membrane by depalmitoylation and then enter the nucleus upon signal induction. (c1-c5) Some proteins release from the membrane by endocytosis and then go to the Golgi. Through Golgi to ER retrograde trafficking, they are finally translocated into the nucleus

Some transmembrane proteins are cleaved within the membrane to release cytosolic segments which are then transported to the nucleus to regulate gene transcription. This mechanism is called regulated intramembrane proteolysis (RIP) [15]. Some proteins are located in the plasma membrane and are processed by RIP to release from the plasma membrane. They consist of extracellular, transmembrane and intracellular subunits. As cell membrane proteins, they are activated by external signals. After proteolysis, their active regions are directly translocated into the nucleus (Fig. 1a1-a2). Here, we provide three examples including Notch, Leukocyte-common antigen-related receptor tyrosine phosphatase (LAR), and amyloid precursor protein (APP).

Notch precursor is first cleaved by a furin-like convertase to generate two subunits within the Golgi apparatus. It is then anchored in the plasma membrane. With the interactions between a transmembrane ligand of the Delta-Serrate Lag family and EGF-like repeats of the extracellular domain of Notch receptors, conformational changes in the receptor expose critical sides for next two steps of cleavage. The second cleavage by a metalloproteinase, a disintegrin and metallop 17 (ADAM17), occurs at the extracellular region near the membrane. The extracelluar N-terminal cleavage products are endocytosed by the signal-sending cell. The third cleavage, mediated by γ-secretase, acts on the intracellular region near the membrane and results in liberation and translocation of the active segment of Notch (NICD, Notch intracellular domain) to the nucleus. In the nucleus, it activates Notch target gene transcription by forming a ternary complex with C-promoter binding factor 1 and the Mastermind-like family of proteins (MAML 1/2/3) [16]. Notch signalling plays a critical role in diverse cellular processes including proliferation, differentiation, and apoptosis. It can also act as an oncogene or tumour suppressor in different tumour subtypes [17–19]. Furthermore, recent research reveals that multipotency of Drosophila intestinal stem cells is regulated by bidirectional Notch signalling. On the one hand, low Notch signalling from a basal enteroendocrine cell to an apical intestinal stem cell blocks enteroendocrine cell differentiation and maintains intestinal stem cell identity. On the other hand, strong Notch signalling from a basal intestinal stem cell to an apical enteroblast promotes enterocyte differentiation [20].

LAR regulates neurite outgrowth and synaptic development by binding to its ligands. The full length LAR is first cleaved by furin to expose its two subunits, which remain noncovalently associated on the plasma membrane. An activating signal results in the α-secretase-mediated second cleavage and release of the extracellular subunit. The third cleavage of the membrane-bound subunit by γ-secretase generates the LAR intracellular domain (LICD). Some LICDs are translocated into the nucleus by interaction with β-catenin and regulate the β-catenin-dependent gene expression [21].

Amyloid precursor protein (APP) undergoes a similar process to that of the Notch and LAR activation pathways, as was presented above. APP is cleaved to generate several fragments including APP intracellular domain (AICD) and the amyloid β peptides. Nuclear AICD is cleaved by β-secretase followed by γ-secretase, while the AICD formed by α-secretase and γ-secretase mainly proceeds to degradation [22]. AICD induces cell death by interacting with Forkhead box (FOX) O in the nucleus upon oxidative stress, and promotes the FoxO-induced pro-apoptotic gene Bim expression as a transcription co-activator [23]. The AICD negatively regulates transcription of Wasf1 and decreases Wasf1 mRNA and protein levels in Neuro 2a cells [24].

#### Depalmitoylation-dependent release from the plasma membrane

There are some transcription factors such as NFAT5a and MfNACsa, which are attached to the plasma membrane by lipid anchors. Upon stimulatory signal, these TFs are depalmitoylated, which releases them from the plasma membrane, and they can then be translocated into the nucleus (Fig. 1b1-b2).

MfNACsa, which belongs to the plant-specific NAC (NAM, ATAF1/2 and CUC2) transcription factors, is a regulator of plant tolerance to drought stress. Under unstressed conditions, MfNACsa is attached to the plasma membrane through palmitoylation, a lipid modification. Upon drought stress, MfNACsa relocates to the nucleus through depalmitoylation mediated by the thioesterase MtAPT1. The nuclear MfNACsa binds the glyoxalase I promoter, resulting in drought tolerance by suppressing glutathione levels [25].

The NFAT5α (nuclear factor of activated T-cells 5, isoform α) transcription factor of *Homo sapiens* is attached to the plasma membrane via both myristoylation and palmitoylation in the resting state. Under osmotic stress conditions, the plasma membrane-bound NFAT5α is transclocated into the nucleus, mediated by reversible palmitoylation but not by proteolytic processing of the lipid-anchored N-terminal region [26].

### Plasma membrane-bound proteins are translocated into the nucleus by Golgi-ER retrograde trafficking

PM-proteins can be released from the plasma membrane by an endocytosis-dependent process. They are internalized by endocytic vesicles (Fig. 1c1) and then are relocated to the Golgi apparatus (Fig. 1c2). They exit from the Golgi through budding (Fig. 1c3), which is mediated by COPI-coated vesicles and are then transported to the endoplasmic reticulum (ER) (Fig. 1c4). Finally, they are transported to the nucleus in full length (Fig. 1c5).

A typical example undergoing this mode of transport is epidermal growth factor receptor (EGFR). EGFR internalization is dependent on binding to its ligand. Different ligands acting on EGFR result in different effects. There are many kinds of EGFR ligands, including TGF-α, β-Cellulin, EGF, HB-EGF, epiregulin, and amphiregulin. EGFR is translocated into the nucleus after stimulation with TGF-α, β-Cellulin, EGF and HB-EGF. These ligands are related to increasing phosphorylation of EGFR tyrosine residues, and induce cell migration. In contrast, both epiregulin and amphiregulin ligands do not result in EGFR nuclear translocation [27].

The endocytosis of EGFR is divided into two categories: clathrin-mediated endocytosis (CME) and nonclathrin endocytosis (NCE) [28–30]. At low EGF concentrations (1 ng/ml), EGFRs are primarily internalized by CME [31]. At high EGF concentrations (20 to 100 ng/ml), EGFR is internalized through CME and NCE. EGFR-NCE is cholesterol and dynamin-dependent, but caveolin-independent, and requires EGFR ubiquitination and proteins harbouring ubiquitin-binding domains [31, 32]. NCE-mediated plasma membrane (PM) invagination processes occurs at ER-PM contact sites with the help of reticulon 3. Local Ca^2+^ release occurs at ER contact sites when tubular invaginations (TI) are formed and is required for the fission of NCE-TI. This Ca^2+^ release process also requires CD147, which is a cargo internalized by NCE [33]. Internalized EGFR is sorted by early endosomes (EE). Some parts of EGFR located in the EE are translocated into the late endosome and are degraded by relocation to the lysosome. Some parts of EGFR located in the EE recycle to the cell surface [34]. Other parts are first translocated into the Golgi membrane and then are transferred to the ER membrane, mediated by COPI-coated trafficking vesicles, and they finally enter the nucleus through the nuclear pore complex [35–37].

EGFR functions as a transcription co-activator with an intrinsic transactivation activity at the C-terminal acidic region. It promotes iNOS gene expression by interaction with transcription factor STAT3 and RNA helicase A. EGFR also promotes cyclin D1 gene expression by interaction with RNA helicase A and promotes the expression of COX-2, Aurora-A and c-Myc genes by interaction with STAT3/5. EGFR induces B-Myb gene expression by interaction with E2F1 [36]. Nuclear EGFR is known to play an important role in tumours. Nuclear transport of EGFR is mediated by vesicular trafficking protein Vps34, and EGFR is recruited to the Arf promoter to repress the transcription of Arf tumour suppressor [38]. Moreover, in renal cell carcinoma, recent evidence indicates that the membranous expression of EGFR has a correlation with poorly differentiated and high nuclear grade tumours, while nuclear EGFR expression is high in well differentiated and low nuclear grade tumours [39].

## The nuclear transportation routes of ER membrane-bound proteins

### ER membrane-bound proteins are translocated into the nucleus directly

#### RUP-dependent process

**Fig. 2:**
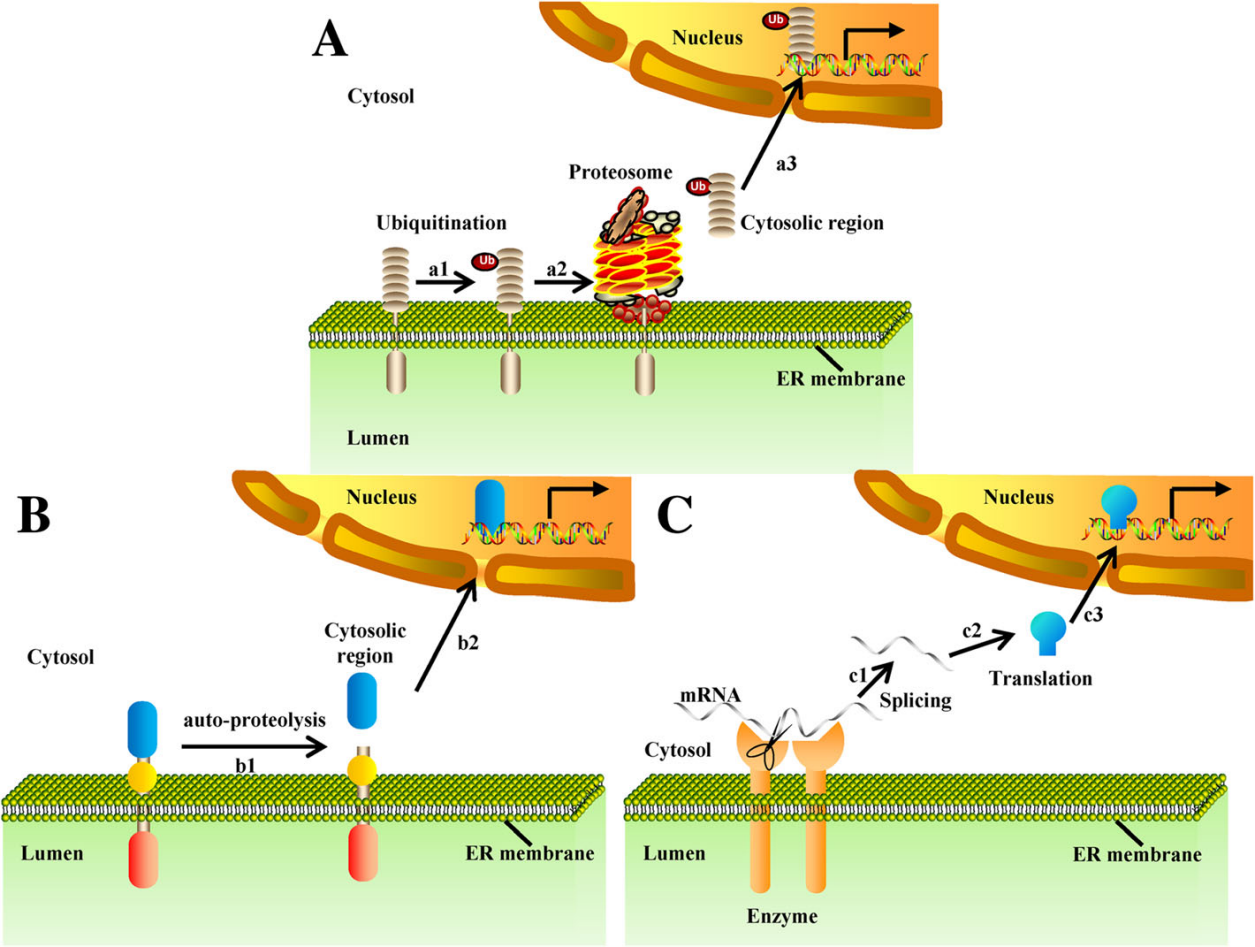
ER membrane-bound proteins are translocated into the nucleus directly. (**a**) Nuclear translocation by RUP. (a1) Proteins get ubiquitinated by ubiquitin ligase. (a2) The ubiquitinated precursor is then processed by a proteasome. The portion within the ER lumen and transmembrane span are degraded by proteasomes. (a3) The active segments enter the nucleus to regulate gene expression. (**b**) Nuclear translocation by auto-proteolysis. (*b1*-*b2*) ER membrane proteins undergo autocatalytic proteolysis, and then the liberated cytosolic fragments are translocated into the nucleus. (**c**) Nuclear translocation by alternative splicing. (c1) Unspliced mRNA transfers to the ER and is spliced by ER enzymes. (c2-c3) Protein fragments translated by spliced mRNA are transported to the nucleus to regulate gene expression

Some ER membrane proteins are translocated into the nucleus directly by regulated ubiquitin/proteasome-dependent processing (RUP). Proteins are ubiquitinated by ubiquitinating enzymes (Fig. 2A, a1), which trigger the ubiquitin/proteasome-dependent degradation process (Fig. 2A, a2). The portions within the ER lumen and transmembrane regions are degraded, leaving the free cytosic segments to enter the nucleus (Fig. 2A, a3).

A yeast transcription factor, suppressor of Ty 23 (SPT23), is regarded as a ER/nuclear membrane-localized dormant precursor and is activated by ubiquitin/proteasome-dependent processing. After ubiquitination of the substrate catalysed by ubiquitin-activating enzyme, ubiquitin-conjugating enzyme, and the RSP5 ubiquitin ligase, the proteasome releases the N-terminal transcription factor domain (p90) by an endoproteolytic reaction. The C-terminal tail is likely degraded by a process that resembles ER-associated degradation [4]. Another study revealed that SPT23 at the ER dimerized via the Ig-like/plexins/transcription factor (IPT) domains before RSP5-mediated monoubiquitination. Processing of SPT23 by the proteasome leads to p90, which is associated with an uncleaved p120 partner molecule. This processing might be assisted by CDC48, ubiquitin fusion degradation 1 (UFD1) and nuclear pore localization 4 (NPL4). The processed p90 retains its ubiquitin modification, and CDC48UFD1/NPL4 removes p90 from its p120 partner. Subsequently, p90 enters the nucleus to drive OLE1 gene transcription [40].

#### Auto-proteolytic-dependent process

Other ER membrane proteins are translocated directly into the nucleus mediated by the auto-proteolytic-dependent releasing mechanism. These proteins have a completely different cleavage mechanism in contrast to RIP or RUP. They undergo an autocatalytic process and are not catalysed by extrinsic proteases. After auto-proteolysis (Fig. 2B, b1), their cytosolic fragments are released and transported to the nucleus directly (Fig. 2B, b2). Here, we provide two examples to illustrate this method.

Myelin regulatory factor (MYRF) is a type-II membrane protein localized on the ER. Trimerization-dependent auto-proteolysis separates its transmembrane domain-containing C-terminal region from its N-terminal fragment. The auto-proteolytic cleavage mechanism has been previously described for the intramolecular chaperone domains of bacteriophage tail-spike proteins. The active N-terminal trimer with two nuclear localization signals is translocated into the nucleus to directly bind the enhancer regions of oligodendrocyte-specific and myelin genes. This signalling also drives synaptic rewiring [41–43].

MrfA, a homologous protein of the metazoan MYRF proteins, is a transcription factor that regulates differentiation of dictyostelium prestalk cells. MrfA is inserted into the ER by its C-terminus-proximal transmembrane (TM) domain. In the ER, the auto-proteolysis of MrfA occurs rapidly and constitutively. The cleavage position lies in its MRF domain, which has extensive similar sequence compared with the C-terminal intramolecular chaperone domain of bacteriophage tail and spike fibre. Additionally, the MRF domain of MrfA contains a serine-lysine dyad that directs its cleavage. The liberated fragment remains cytosolic in growing cells, while the liberated fragment is activated and accumulated in the nucleus in some anterior-like cells and prestalk cells. Due to the regulated nuclear translocation of the liberated fragment, MrfA has a role in prestalk cell differentiation [9].

#### Alternative splicing-dependent process

Still other ER membrane-bound proteins are translocated into the nucleus directly, mediated by the alternative splicing-dependent mechanism. With stimuli such as ER stress, unspliced mRNA goes to the ER and is spliced by an ER enzyme (Fig. 2C, c1). Protein fragments translated by splicing mRNA (Fig. 2C, c2) are transported to the nucleus to regulate gene expression (Fig. 2C, c3).

The protein bZIP60 encoded by unspliced mRNA is predicted to be a type II membrane protein in the ER with a single transmembrane domain. In maize, the segment of mRNA encoding bZIP60 is folded into a twin loop structure. In response to ER stress, the mRNA is spliced by cleaving a 20b intron by a membrane-associated dual-functioning protein kinase/ribonuclease known as inositol-requiring enzyme 1 (IRE1). Splicing transforms the predicted protein from a membrane-associated transcription factor to the active bZIP transcription factor, which is then targeted to the nucleus. In maize seedlings, bZIP60 splicing is initiated by ER stress agents such as tunicamycin or dithiothreitol or by heat treatment [44].

X-box-binding protein 1 (XBP1) is also known to be activated by a splicing-dependent mechanism. XBP1 is activated by ER membrane enzyme-mediated splicing. Under ER stress conditions, unspliced XBP1 messenger RNA precursor (XBP1u mRNA) is spliced on the ER membrane by IRE1. After translation, active transcription factor XBP1s (the spliced form of XBP-1) goes to the nucleus to alleviate ER stress [45]. After partial hepatectomy, XBP1 enters the nucleus to induce STAT3 transcription. Decreased XBP1 levels promote DNA damage responses in regenerating hepatocytes [46].

### ER membrane-bound proteins are translocated into the nucleus by trafficking to the Golgi

**Fig. 3:**
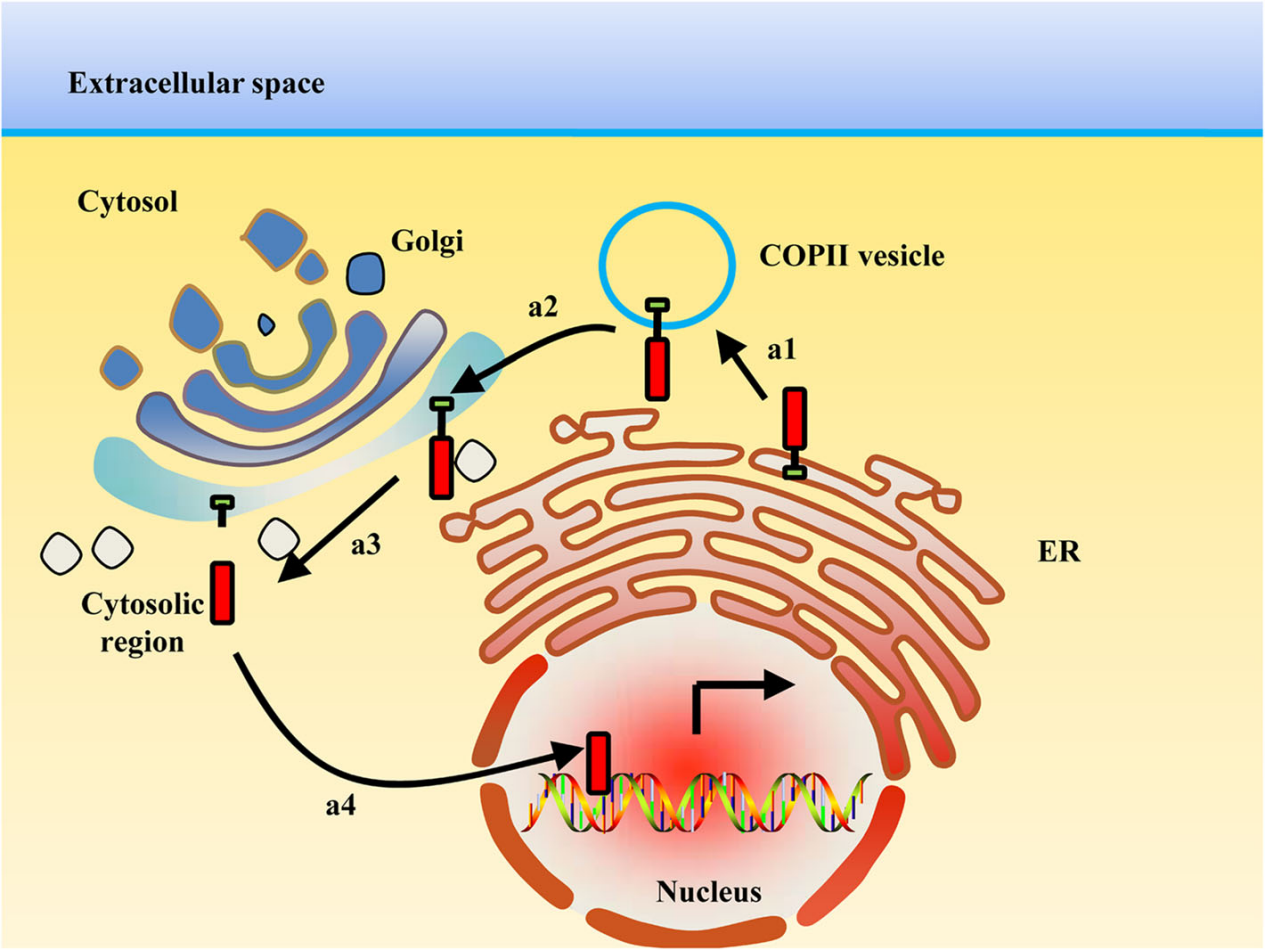
ER membrane-bound proteins relocate in the nucleus by trafficking to the Golgi. (a1-a2) ER membrane-located proteins are transported to the Golgi by COPII vesicles. (a3) Within the Golgi apparatus they are cleaved by Golgi-resident proteases. (a4) Finally, soluble cytosolic segments enter the nucleus to play a transcription regulation role

Some proteins are first located in the ER membrane and are activated upon ER stress. When activated, these proteins leave the ER and are transferred to the Golgi by COPII-mediated trafficking vesicles (Fig. 3a1-a2). Within the Golgi apparatus, they are cleaved by Golgi-resident proteases (Fig. 3a3). Finally, the soluble cytosolic segments enter the nucleus to exert a transcription regulation role (Fig. 3a4). Examples are provided to illustrate this mode, including sterol regulatory element-binding protein (SREBP), plant bZIP TF family protein bZIP28 and several activating transcription factor (ATF)/cAMP response element-binding protein (CREB) family proteins, such as activating transcription factor 6 (ATF6), old astrocyte specifically induced substance (OASIS) and spinal cord injury and regeneration-related protein #69 (SCIRR69).

For SREBP, recent research demonstrates that EGFR signalling increases glucose uptake, and then promotes N-glycosylation of SREBP cleavage-activating protein (SCAP). Glycosylation promotes the stability of SCAP, reduces linkage between insulin-induced gene 1 and SCAP, and makes SCAP/SREBP move from the ER to the Golgi. Activated N-terminal fragments generated by protein enzyme cleavage enter the nucleus and promote tumour growth by regulating the expression of lipogenesis genes [47]. Structural analysis on endoplasmic reticulum membrane–anchored sterol sensors, Insigs, reveals that they form a V-shaped cavity with their six transmembrane segments (TMs). TM3 and TM4 are responsible for Insig-2 binding to SCAP [48].

The plant basic leucine zipper (bZIP) transcription factor, bZIP28, is a type II membrane protein which has a single transmembrane domain. Its N-terminal fragment faces the cytosolic side, and its C-terminal fragment faces the ER lumen side. bZIP28 interacts with binding immunoglobulin protein (BiP) in the ER lumen via its C-terminal domain. The release of BiP from bZIP28 is responsible for ER-Golgi trafficking of bZIP28 under stress conditions [49]. It has been extensively recognized that the activation of bZIP28 is achieved by site-1 and site-2 protease, which are Golgi-resident proteases. However, a recent study reveals that bZIP28 cleavage is mediated by site-2 protease, but not by site-1 protease [50].

As to ATF6, dissociation of BiP from ATF6 is also important for its ER-Golgi translocation. ATF6 is known to induce the expression of ER stress response genes under ER stress conditions. However, ATF6 induces the expression of numerous oxidative stress response genes after ischaemia/reperfusion [51]. Upon bacterial infection, ATF6 is also critical for regulating interferon gamma-induced Dapk1 expression by association with CCAAT/enhancer-binding protein beta. Without ATF6, interferon gamma fails to induce autophagy in cells. This result links ER stress and autophagy [52].

OASIS is a basic leucine zipper (bZIP) transcription factor which belongs to the CREB/ATF family. During ER stress, it is notable that OASIS is not induced at the transcriptional level in any other cell types examined but only in astrocytes of the central nervous system, which exhibit cell type-specific unfolded protein response signalling [53].

SCIRR69 is located in the cytoplasm of primary neurons under normal conditions, but it is transferred to the nucleus via shedding from the membrane upon neuron damage [12]. SCIRR69 regulates BDNF gene expression [54]. SCIRR69-dependent signalling involves protein interactions with sideroflexin-1 and transitional endoplasmic reticulum ATPase [55].

## Conclusion

Compared with common transcription factors, MTFs obviously belong to a special class of transcription factors. Their movement ability is limited until activated by stimuli. The release of dormant MTFs provides a means for rapid responses to external and internal stimuli, and they are considered to play important roles in signalling upon various cues and stresses. As mentioned above, MTFs play critical roles in environmental stress and cellular ER stress responses. They help plants to cope with unfavourable growth conditions by regulating gene expression with their transcriptional activity [56]. Extending our understanding of the molecular mechanisms underlying how intracellular movement of MTFs is organized may provide us with an advantage by enabling plants to increase their stress tolerance. Moreover, there are also some MTFs which are involved in the development and progression of tumours, such as Notch, EGFR and SREBP. Examination of the subcellular locations where they are processed and of their related functional proteins may provide potential molecular targets for designing novel therapeutic drugs. Nuclear translocation regulation of MTFs occurs through a series of events. Targeting the modulation of membrane properties, spatial structure of membrane proteins and the corresponding processes of proteases could potentially transform nuclear transport routes such as endocytosis or ER-Golgi trafficking. This may significantly improve the survival of patients, for example, γ-secretase inhibitors (GSIs) suppress tumour growth in several preclinical cancer models by blocking the cleavage of Notch at the cell membrane, effectively inhibiting the release of the active NICD subunit [57–59]. Studies have shown that patients with high nuclear EGFR levels have poor clinical outcomes in breast cancer [60], which implies that nuclear EGFR may benefit tumours by helping to evade cell surface EGFR-targeted small molecule inhibitors and therapeutic antibodies. Therefore, inhibition of EGFR nuclear translocation may increase overall survival of patients. Collectively, targeting intracellular movement pathways of MTFs is valuable for therapeutics, and further research will help us to find more effective treatments.

Taken together, MTFs and their nuclear translocation are recently gaining more attention and this is an expanding research area. Further development in this area provides an effective complement to the mechanisms of signal transduction and gene regulation. Studying nuclear transportation routes of MTFs provides a new idea for drug design.

## Abbreviations

ADAM17: A disintegrin and metallop 17
AICD: APP intracellular domain
APP: Amyloid precursor protein
ATF: Activating transcription factor
BiP: Binding immunoglobulin protein
bZIP: basic leucine zipper
CME: Clathrin-mediated endocytosis
CREB: cAMP response element-binding protein
EE: Early endosome
EGFR: Epidermal growth factor receptor
ER: Endoplasmic reticulum
FOX: Forkhead box
GSIs: γ-secretase inhibitors
IPT: Ig-like/plexins/transcription factors
IRE1: Inositol-requiring enzyme 1
LAR: Leukocyte-common antigen-related receptor tyrosine phosphatase
LICD: LAR intracellular domain
MTFs: Membrane-bound transcription factors
MYRF: Myelin regulatory factor
NCE: Nonclathrin endocytosis
NFAT5α: Nuclear factor of activated T-cells 5, isoform α
NICD: Notch intracellular domain
NPL4: Nuclear pore localisation 4
OASIS: Old astrocyte specifically induced substance
PM: Plasma membrane
RIP: Regulated intramembrane proteolysis
RUP: Regulated ubiquitin/proteasome-dependent processing
SCAP: SREBP cleavage-activating protein
SCIRR69: Spinal cord injury and regeneration-related protein #69
SPT23: Suppressor of Ty 23
SREBP: Sterol regulatory element-binding protein
TFs: Transcription factors
TI: Tubular invaginations
TM: Transmembrane
UFD1: Ubiquitin fusion degradation 1
XBP1: X-box-binding protein 1
